
JOURNAL INFORMATION
==============================
NLM Title Abbreviation: ScientificWorldJournal
Journal ID: ScientificWorldJournal
NLM Journal ID: 101131163
Journal ID: TSWJ

The Scientific World Journal

ISSN: 2356-6140
EISSN: 1537-744X
Publisher: Wiley

ARTICLE INFORMATION
==============================
PMCID: PMC6009237
PMID: 29973811
DOI: 10.1100/tsw.2002.37
Article ID: 158189
Article version: 1
Subjects: Short Report

Characterize Protein Functional Relationships Based on Mrna Expression Profile

Ding Wei *
Wang Luquan
Qiu Ping
Greene Jonathan
Hernandez Marco
Bioinformatics Group and Human Genomics Research Division at Schering-Plough Research Institute, 2015 Galloping Hill Road, Kenilworth, NJ 07033, UK
*Wei Ding: wei.ding@spcorp.com
Publication date: 2002
Electronic publication date: 2002 Feb 23
Volume: 2
First page: 77
Last page: 78
Copyright: Copyright © 2002 Wei Ding et al.
Copyright year: 2002
License: This is an open access article distributed under the Creative Commons Attribution License, which permits unrestricted use, distribution, and reproduction in any medium, provided the original work is properly cited.
License URL: https://creativecommons.org/licenses/by/3.0/

==============================
Advances in Gene Technology: The Genome and Beyond –
Structural Biology for Medicine (Proceedings of the 2002 Miami
*Nature Biotechnology* Winter Symposium)
*TheScientificWorld* 2002, 2(S2), 77–78
ISSN 1532-2246; DOI 10.1100/tsw.2002.37

### CHARACTERIZE PROTEIN FUNCTIONAL RELATIONSHIPS BASED ON MRNA EXPRESSION PROFILE

Wei Ding, Luquan Wang, Ping Qiu, Jonathan Greene, and Marco Hernandez
Bioinformatics Group and Human Genomics Research Division at Schering-Plough Research Institute, 2015 Galloping Hill Road, Kenilworth, NJ 07033
wei.ding@spcorp.com

**INTRODUCTION**. Protein families are distinguished by members that exhibit sequence and biomedical function similarity. For most gene changes in protein abundance are related to changes in mRNA abundance, which are immensely informative about cell state and the activity of genes. LifeExpress RNA (LE) database (Incyte Genomics, Inc) is a large-scale genome expression database. Based on LE we derived the functional relationship of Pfam[1], a database of protein domain families, by studying the global expression profile of the corresponding genes of Pfam family members. The expression profiles for 135 largest Pfam families were summarized and relationships were analyzed. The study present a simple model for conceptualizing the complex genetic regulatory network.

**METHOD**. We used the BLAST search algorithm[2] to match Pfam family members to the Incyte clones on LE. 4177 Incyte clones were mapped to 1069 Pfam families. 555 LE was averaged by the mean for repeated experiments, and each expression datum was then reduced to a binary variable (regulated or unregulated). Family Regulation Ratio (FRR), which represents the regulated member percentage, was assigned to the Pfam family for each probe pair. In order to reduce the random noise, we only study those Pfam families with more than 10 clones and include 135 families in our data analysis. The 555 probe pairs were also randomly divided into two test data sets for the validation purpose. Two test expression profiles for each Pfam were computed.

**RESULTS.** Pearson correlation coefficients (PCC) were calculated between every two expression profiles of Pfam families with shared clones excluded. There are 79 Pfam pairs with PCC >= 0.75 (Table1). PCC1 and PCC2 were also computed for two test data sets, respectively, and were correlated very well, which validated our approach.

**DISCUSSION.** We present a simple model for conceptualizing how gene/protein families interact to generate a complex and robust system. Boolean network models are based on a binary idealization of gene expression levels. The choice of threshold is arbitrary. Although this model is oversimplified, abstraction may be useful in conceptualizing the nature of genetic information flow. Many of those protein family relationships identified here are supported by functional link among those families. Taking G protein-coupled receptors (GPCRs) as an example, after agonist action, GPCRs activate G proteins and become phosphorylated by G protein-coupled receptor kinases[3]. This event further promotes activation of effector enzymes and ion channels by the activated GαGTP. This GPCR signal transduction pathway is reflected in our study by the strong correlation between 7 transmembrane receptors (rhodopsin family) and protein kinases, and that between rhodopsin family and ion transport proteins. This study also revealed many intriguing links between protein families, which provided novel hypothesis for further functional study.

Table 1. Lists the Pfam family pairs with the highest PCC.

| *PfamID1* | *Description* | *Clone Num* | *PfamID2* | *Description* | *Clone Num* | *Correlation* |
|---|---|---|---|---|---|---|
| PF00001 | 7 transmembrane receptor (rhodopsin family) | 124 | PF00069 | Protein kinase domain | 231 | 0.819 |
| PF00001 | 7 transmembrane receptor (rhodopsin family) | 124 | PF00520 | Ion transport protein | 49 | 0.816 |
| PF00008 | EGF-like domain | 78 | PF00041 | Fibronectin type III domain | 75 | 0.836 |
| PF00008 | EGF-like domain | 79 | PF00069 | Protein kinase domain | 230 | 0.824 |
| PF00036 | EF hand | 66 | PF00069 | Protein kinase domain | 231 | 0.802 |
| PF00041 | Fibronectin type III domain | 77 | PF00096 | Zinc finger, C2H2 type | 190 | 0.805 |
| PF00046 | Homeobox domain | 71 | PF01094 | Receptor family ligand binding region | 16 | 0.814 |
| PF00046 | Homeobox domain | 71 | PF00595 | PDZ domain (Also known as DHR or GLGF). | 53 | 0.8 |
| PF00069 | Protein kinase domain | 223 | PF00169 | PH domain | 67 | 0.814 |
| PF00069 | Protein kinase domain | 228 | PF00560 | Leucine Rich Repeat | 62 | 0.81 |
| PF00069 | Protein kinase domain | 231 | PF00105 | Zinc finger, C4 type (two domains) | 32 | 0.805 |
| PF00069 | Protein kinase domain | 231 | PF00104 | Ligand-binding domain of nuclear hormone receptor | 32 | 0.802 |
| PF00096 | Zinc finger, C2H2 type | 190 | PF00595 | PDZ domain (Also known as DHR or GLGF). | 53 | 0.801 |
| PF00096 | Zinc finger, C2H2 type | 190 | PF00520 | Ion transport protein | 49 | 0.8 |

**REFERENCES**

1. Bateman, A., Birney, E., Durbin, R., Howe, K., and Sonnhammer, E.L. (2000) Nucl. Acids Res.28, 263–266.
2. Altschul, S.F., Gish, W., Miller, W., Myers, E.W., and Lipman, D.J. (1990) J. Mol. Biol. 215, 403–410.
3. Lefkowitz, R.J. (1998) J. Biol. Chem. 273, 18677–18680.
